# Impact of on-trial IGRT quality assurance in an international adaptive radiotherapy trial for participants with bladder cancer

**DOI:** 10.1016/j.radonc.2024.110460

**Published:** 2024-10

**Authors:** Amanda Webster, Michael Francis, Hannah Gribble, Clare Griffin, Shaista Hafeez, Vibeke N. Hansen, Rebecca Lewis, Helen McNair, Elizabeth Miles, Emma Hall, Robert Huddart, on behalf of the RAIDER Trial Management Group

**Affiliations:** a National Radiotherapy Trials Quality Assurance (RTTQA) Group, University College Hospital (UCLH), 235 Euston Road, London NW1 2BU, United Kingdom; b The Royal Marsden NHS Foundation Trust, Downs Road, Sutton SM2 5PT, United Kingdom; c Clinical Trials and Statistics Unit, The Institute of Cancer Research, 15 Cotswold Road, London SM2 5NG, United Kingdom; d Division of Radiotherapy and Imaging, The Institute of Cancer Research, 15 Cotswold Road, London SM2 5NG, United Kingdom; e Copenhagen University Hospital – Rigshospitalet, Department of Oncology, Blegdamsvej 9, 2100 København, Denmark; f National Radiotherapy Trials Quality Assurance (RTTQA) Group, Mount Vernon Hospital, Rickmansworth Road, Northwood HA6 2RN, United Kingdom

**Keywords:** IGRT, Adaptive, Bladder cancer, Plan selection, Quality assurance

## Abstract

**Background and purpose:**

Radiotherapy trial quality assurance (RT QA) is crucial for ensuring the safe and reliable delivery of radiotherapy trials, and minimizing inter-institutional variations. While previous studies focused on outlining and planning quality assurance (QA), this work explores the process of Image-Guided Radiotherapy (IGRT), and adaptive radiotherapy.

This study presents findings from during-accrual QA in the RAIDER trial, evaluating concordance between online and offline plan selections for bladder cancer participants undergoing adaptive radiotherapy. RAIDER had two seamless stages; stage 1 assessed adherence to dose constraints of dose escalated radiotherapy (DART) and stage 2 assessed safety. The RT QA programme was updated from stage 1 to stage 2.

**Materials and methods:**

Data from all participants in the adaptive arms (standard dose adaptive radiotherapy (SART) and DART) of the trial was requested (33 centres across the UK, Australia and New Zealand). Data collection spanned September 2015 to December 2022 and included the plans selected online, on Cone-Beam Computed Tomography (CBCT) data. Concordance with the plans selected offline by the independent RT QA central reviewer was evaluated.

**Results:**

Analysable data was received for 72 participants, giving a total of 884 CBCTs. The overall concordance rate was 83% (723/884). From stage 1 to stage 2 the concordance in the plans selected improved from 75% (369/495) to 91% (354/389).

**Conclusion:**

During-accrual IGRT QA positively influenced plan selection concordance, highlighting the need for ongoing support when introducing a new technique. Overall, it contributes to advancing the understanding and implementation of QA measures in adaptive radiotherapy trials.

## Highlights

• First international bladder radiotherapy trial to assess tumour-focused adaptive radiotherapy.
• Novel IGRT quality assurance programme implemented and reviewed from stage 1 to stage 2 of the trial.
• Appropriateness of plans selected online reviewed centrally offline.
• Concordance of plans selected online and centrally improved, 75% for stage 1 to 91% for stage 2.

## Introduction

Radiotherapy trial quality assurance (RT QA) plays a crucial role in ensuring the safe and reliable delivery of radiotherapy trials. Its primary objective is to mitigate inter-institutional variations in equipment, practices, software, and staff expertise that could potentially bias and invalidate trial results. In the United Kingdom, the National Radiotherapy Trials Quality Assurance (RTTQA) Group, comprising multidisciplinary experts, monitors adherence to a trial protocol in conjunction with the trials unit. The group aims to minimise variations, ensuring clinical trial outcomes reflect differences in randomised treatment allocations rather than departures from the trial protocol [1].

In general, RT QA in trials encompasses comprehensive reviews of pre-treatment imaging, outlining, planning, and dosimetry audits [2]. It is a resource-intensive process for both institutions and the quality assurance group but a range of studies have demonstrated the significant benefits of RT QA in trials [3], [4], [5], [6], [7], [8], [9], [10], [11], [12]. In general, these studies have focused on the quality assurance of the outlining and planning phases of the patient's radiotherapy pathway and there has been, to date, less of a focus on the process of Image-Guided Radiotherapy (IGRT), particularly adaptive radiotherapy. Generally, QA can be conducted either pre-accrual i.e., before the centre opens the trial, and/or during-accrual.

With the increasing complexity of radiotherapy techniques, the process of delivering radiotherapy has become more intricate, prompting a closer examination of IGRT QA. For Therapeutic Radiographers/Radiation Therapists (RTT), 3D IGRT allows for more sophisticated decision-making than only geometrically verifying the treatment position. Adaptive radiotherapy such as the library of plans/plan of the day (PoD) approach gives further opportunity to extend the roles of the RTT. The aim of PoD approach is to predict and assess the anatomical changes that occur, e.g., bladder filling, and select plans to compensate for these changes [13]. This approach was studied in the RAIDER randomised controlled two-stage trial (NCT02447549), which evaluated the use of small, medium, and large plans, produced by adding varying PTV expansions, to treat bladder cancer participants [14] and also had the additional complexity of delivering two dose levels to the bladder (Appendix A).

Consequently, all RTTs involved in RAIDER underwent intensive pre-accrual training to accredit them to undertake POD selections for participants treated in the trial [15], [16]. This training, delivered by the RTTQA Group in collaboration with the RAIDER Trial Management Group (TMG) and Trans-Tasman Radiation Oncology Group (TROG) cancer research, comprised workshops, videos, workbooks, training cases, and test cases; 508 RTTs successfully completed the training. To be accredited RTTs needed to pass a competence test, agreeing with an expert consensus standard on > 83 % of matches; 461/508 (91 %) of RTTs passed on their first attempt, 39/508 (8 %) on their second and 8/508 (1 %) on their third attempt [15]. The past rate of 83 % was chosen based on the limited literature available at the time of developing the QA programme, and consensus of the TMG [17]. However, there is no evidence to support the sufficiency of pre-accrual IGRT training only, leading the RAIDER team to undertake quality assurance during-accrual. Cone Beam CTs (CBCTs) acquired on participants enrolled in the trial were collected, and the plans selected by RTTs online were independently reviewed centrally offline. This process ensured the quality assurance of online plan selections, fulfilling an exploratory trial endpoint; to review the appropriateness of the plans selected. This work presents the findings from the during-accrual QA, including an overview of the selected plans and the concordance between online and offline central plan selections in both stages of the RAIDER trial.

## Methods

### Population

The recruitment process took place in the United Kingdom, Australia, and New Zealand, in total 33 centres completed the RT QA programme and enrolled participants. Analysis presented here includes participants who were randomised into either of the adaptive arms of the trial, namely SART (standard-dose adaptive radiotherapy) and DART (dose-escalated adaptive radiotherapy) [14]. Participants assigned to the control arm were excluded as they did not receive adaptive radiotherapy. Participants recruited in both stage 1 (assessing adherence to dose constraints of DART) and stage 2 (assessing safety) of the trial are included.

### Quality assurance of selected plans

During the transition from stage 1 to stage 2, a central review was undertaken by RTTQA (AW) and the plans selected online by participating centres were reviewed centrally, to ensure compliance with trial guidelines. These central reviews were conducted on an ongoing basis as the trial continued to recruit participants and centres encountered challenges in delivering tumour-focused PoD radiotherapy [16]. Previously we have described updates to the QA program following this analysis of stage 1 participants, including a workshop, additional guidance on case scenarios, flow diagrams, step-by-step instructions, and one-to-one sessions [15]. Additionally, we introduced plan selection case reports during the accrual phase, (Appendix B). These reports were used to communicate to the centres whether their selected treatment plans were compliant with the guidelines or not. Guidance was given if PoD selections were non-compliant and additional reviews were undertaken for further recruited participants.

### Ethics

The Clinical Trials and Statistics Unit at the Institute of Cancer Research (ICR-CTSU) obtained approval for the RAIDER study from the London-Surrey Borders Research Ethics Committee (15/LO/0539). All RAIDER participants provided informed consent to take part in the trial.

### Data collection

Data collection occurred between September 2015 and December 2022, during which RTTQA centrally gathered Digital Imaging and Communications in Medicine (DICOM) data, including CT planning scans, RT structure set, three RT plans and the associated RT dose cubes. Plan selection forms (PSFs) and a minimum of weekly pre- and post-CBCTs, all registered in the treatment position were also included. Demographic data and disease characteristics were reported to ICR-CTSU by participating sites. To ensure confidentiality, all data, underwent pseudo anonymization and were securely stored on a protected network computer. The data for all adaptive patients was requested. The aim was to review the data for at least 1 adaptive participant from centres that recruited into the RAIDER trial. Once this was achieved any additional data that had been sent correctly was added to this evaluation.

### Data analysis

The plans selected online were reviewed, using the PSF and CBCTs. The online adaption rate (i.e., the number of small, medium and large plans) was assessed, for stage 1 and stage 2 participants separately. Next, the CBCTs and plans selected were independently reviewed centrally by AW. The offline central adaption rate was assessed for stage 1 and stage 2 participants. A comparison of the difference in level of agreement for stage 1 and 2 was done using a test of proportion. For the offline adaption rate if more than one plan was suitable for selection the smallest suitable selection was analysed. The agreement between the online (as treated) and offline central selections was evaluated as part of the during-accrual QA. For the purpose of during-accrual QA in cases where multiple plans were centrally deemed suitable for treatment (when balancing the different aspects of conformance, such as bladder filling status, dose to tumour and surrounding healthy structure, e.g. bowel), we considered there to be agreement if at least one option aligned with the decision made online. After recruitment completed a cross-tabulation of the online and offline selections was produced. This tabulation includes the details of cases when more than one plan/action was deemed suitable in the offline central review. A random subset (5 %) of the CBCTs and plans selected by the first author were also blindly reviewed centrally, by a second independent reviewer (MF) to ensure consistency with guidelines.

The data were analysed using SPSS version 29.

## Results

**Fig. 1:**
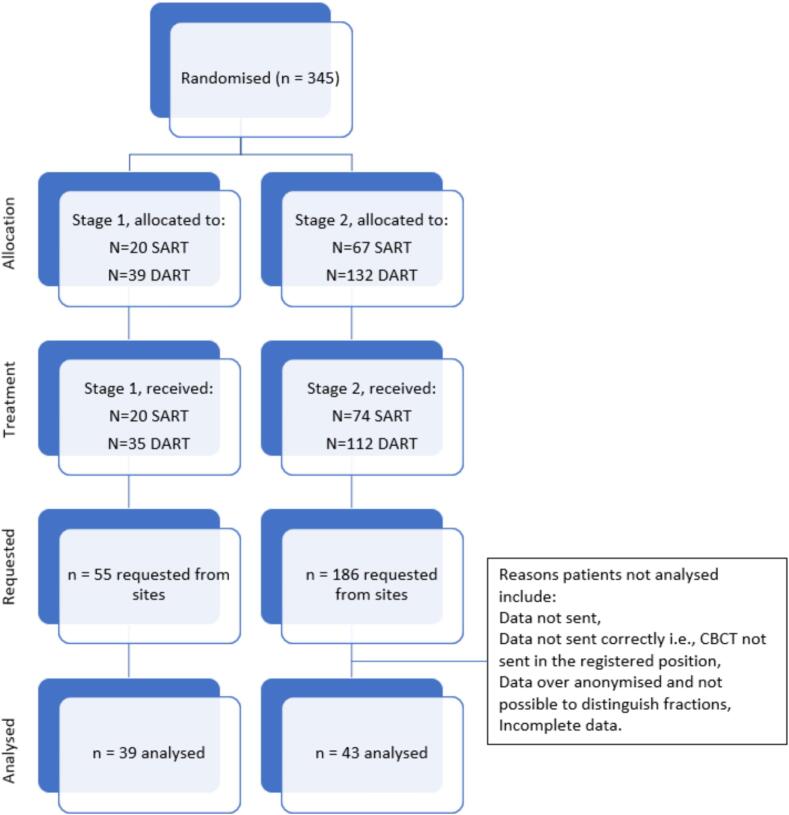
Consort diagram of patients included in analysis.

Analysable DICOM data was received for eighty-two participants treated with adaptive radiotherapy, which resulted in 884 CBCTs being centrally reviewed (Fig. 1). From two (0.2 %) of the CBCTs the treatment could not be delivered to the participant and therefore the adaption rates were calculated from 882 CBCTs. Thirty-nine of the participants reviewed were recruited in stage 1 and 43 participants in stage 2. Forty-three participants received DART treatment and 39 participants received SART treatment.

The online adaption rates are reported for stage 1 and 2. In stage 1, 47/485 (10 %) of plans selected online were small, 203/485 (42 %) were medium, 208/485 (43 %) were large. In a further 27/485 (5 %) cases the participant was removed from the treatment couch: 8 cases when the bladder was too full for the large plan to be selected, 7 cases when the bladder was too small and the tumour was not in the high-dose treatment volume, 3 cases with too much rectum in the treatment volume, specifically the high-dose treatment volume, 4 cases when the RTTs did not think the small or medium plan was suitable, 2 cases when the participant was not able to maintain a full bladder, and 3 cases when the reasons were not provided.

**Fig. 2:**
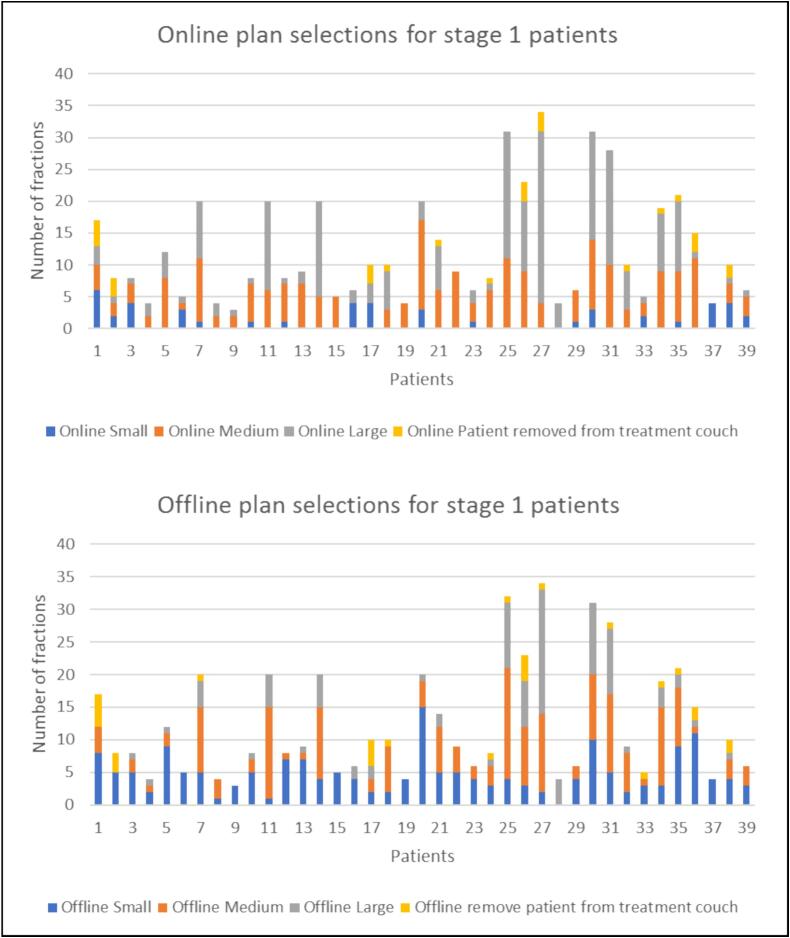
Online and offline plan selections for stage 1 patients.

**Fig. 3:**
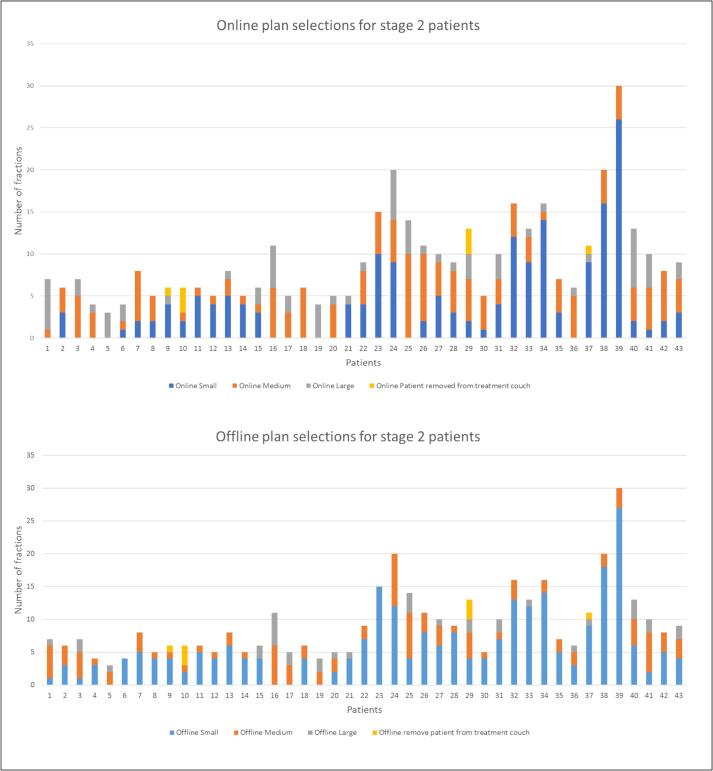
Online and offline plan selections for stage 2 patients.

In stage 2, 176/397 (44 %) of plans selected online were small, 145/397 (37 %) were medium, 68/397 (17 %) were large and in 8/397 (2 %) of cases the participant was removed from the treatment couch (4 cases when the bladder was too small and the tumour was not in the high-dose treatment volume, 2 cases when the bladder was too full for the large plan to be selected and 2 cases when the reasons were not provided). The online plan selections for stage 1 and stage 2 participants is presented in Fig. 2, Fig. 3.

For the offline central adaption, in stage 1, 183/485 (38 %) of plans selected centrally were small, 177/485 (36 %) were medium, 96/485 (20 %) were large and in 29/485 (6 %) of cases it was recommended to remove the participants from the treatment couch. In stage 2, 253/397 (64 %) of plans selected centrally were small, 101/397 (25 %) were medium, 35/397 (9 %) were large and in 8 (2 %) of cases it was recommended to remove the participants from the treatment couch. Offline and online reasons for removing the patient from the treatment couch were the same with two additional instances when the offline central review would have recommended removal of the patient from the treatment couch, where the bladder was too small and where the tumour was not encompassed by the high-dose treatment volume. The offline plan selections for stage 1 and stage 2 participants are presented in Fig. 2, Fig. 3. Five-percent of central selections made by the first author were reviewed by a second independent reviewer. All plan selections between the first and second central reviewers were concordant.

On-trial, the overall rate of concordance between the plans selected online with the offline central review was 83 % (723/884). In stage 1, 75 % (369/495) of plans selected were compliant with the offline selection and in stage 2, 91 % (354/389) of plans selected were compliant with the offline selection. The improved compliance between stage 1 and stage 2 was statistically significant, p < 0.001, Table 1.

**Table 1:** Frequency of agreement in plan selections.

	**Stage**	**Agree**	**Frequency**	**Percentage**	**p value**
n = 495	**Stage 1**	Yes	369	74.50 %	p < 0.001
	**Stage 1**	No	126	25.50 %	
n = 389	**Stage 2**	Yes	354	91.00 %	
	**Stage 2**	No	35	9.00 %	

*882 plus 2 fractions when the patient did not receive treatment.

Following the completion of recruitment, we generated a cross-tabulation comparing online and offline selections, Table 2. This detailed tabulation highlights instances where the offline central review identified multiple suitable plans or actions.

**Table 2:** Post-accrual comparison of online and offline plan selections including details of when more than one plan/action suitable.

Offline central selection										
		Small/Remove from couch	Small	Medium/Small	Medium	Medium/Remove from couch	Medium/Large	Large	Large/Remove from couch	Remove from couch
Online selection	Small	9 (1%)	202 (23%)	6 (<1%)	2 (<1%)	0	0	0	0	4 (<1%)
	Medium	0	82 (9%)	119 (13%)	139 (16%)	1 (<1%)	4 (<1%)	3 (<1%)	0	0
	Large	0	3 (<1%)	14 (2%)	54 (6%)	1 (<1%)	76 (9%)	121 (14%)	4 (<1%)	3 (<1%)
	Remove patient from couch	0	0	1 (<1%)	0	0	0	3 (<1%)	1 (<1%)	30 (3%)

## Discussion

During-accrual IGRT trial QA appears to improve the plan selection concordance, in an international adaptive radiotherapy trial for participants with bladder cancer. The overall rate of concordance between online and central selections was 83 %. In stage 1 there was a modest concordance rate of 75 % in the plan selected online with the plans selected centrally offline. There was bias towards the online selection being larger than the central reviewer’s selection. This results in the small plan being selected at fewer fractions and large more frequently than was considered optimal. The low concordance rate represented a risk of missing the possible benefit of the experimental treatment technique and highlighted the need for further quality assurance and support. A concordance rate of 83 % in the pre-accrual training was considered as a pass and the stage 1 concordance rate was worse than this.

The introduction of during-accrual RT QA measures [15] led to a significant improvement in concordance of plan selection. Notably, in stage 2 of the trial, the concordance rate increased to 91 % with many more fractions delivered by the appropriate plan selection. The small plan was more often chosen when suitable and there were less large plan selections. Additionally, the participants appeared to be removed from the treatment couch less often in stage 2 compared to stage 1. Plan selections and removing the participant from the treatment couch was a topic that was discussed at length during a workshop with centres and alternative interventions and solutions were explored [15]. This was to enhance the participants comfort by reducing the need to remove them from the treatment couch. It can also have a positive impact on the workflow in departments. These findings emphasize the importance of implementing rigorous during the accrual QA in complex radiotherapy trials [15], [16].

The novelty of this work lies in its comprehensive examination of IGRT QA in an adaptive trial, which included delivering the treatment to multiple dose levels and dose escalation. Given the intricate nature of the radiotherapy procedures within the trial, treatment centres naturally expressed concerns upon initiating the trial, particularly regarding potential repercussions for participants and workflow efficiency. Notably, among the reviewed CBCTs there were only 35 (4 %) instances when the participant had to be removed from the treatment couch. This finding indicates there was minimal impact on participants and resources in delivering multiple dose levels to bladder cancer participants.

To the best of our knowledge, no previous study has undertaken such a comprehensive investigation in the context of adaptive radiotherapy trials. By studying the concordance between online and central plan selections, this research provides valuable insights into the effectiveness of IGRT QA in ensuring guideline compliance and consistency across multiple centres. The inclusion of during-accrual plan selection case reports further enhances the novelty of this study, as these reports have previously been utilised solely for the outlining and planning RT QA. They played a critical role in facilitating communication between centres and the QA group, enabling real-time feedback and guidance to ensure the quality and adherence of plan selections. This study fills a critical gap by examining IGRT QA in adaptive radiotherapy trials, which has been overlooked in previous studies focusing on outlining and planning [3], [4], [5], [6], [7], [8], [9], [10], [11], [12]. IGRT QA is essential for ensuring accurate and precise treatment delivery, particularly in complex radiotherapy techniques. The improved concordance underscores the value of during-accrual RT QA despite its resource-intensive nature.

With regards to the staff who can deliver adaptive radiotherapy, trained RTTs are capable of safely delivering adaptive radiotherapy. In total, 508 RTTs were trained and approved through the RAIDER PoD QA programme. With appropriate training, guidance, and support, they play a crucial role in successfully implementing adaptive radiotherapy trials. This work however highlights that to attain best standards QA needs to be considered as an iterative process with a role for ongoing real-time feedback. For the RAIDER trial, a pragmatic training approach had to be utilised that was applicable for a multicentre trial. However, ideally training needs should be addressed before IGRT QA commences [18]. Nonetheless, this study serves as a novel example of the potential of RTTs in delivering adaptive radiotherapy and highlights the significance of incorporating IGRT QA into radiotherapy trials.

Further review of the reasons a participant was removed from the treatment couch can shed light on whether there is a need for real-time adaption when treating bladder participants. There were only 2 occasions in this study when treatment could not be delivered. There were a further 35 occasions when the participant had to be removed from the treatment couch, but the treatment was delivered on the second attempt. Real-time adaption might have been beneficial in these 4 % of cases, though the cost-effectiveness aspect warrants careful consideration. The post-accrual comparison of online and offline selections further sheds light on the fact that more than one plan/action could have been suitable for the participant and in ∼ 37 % of cases at least two actions were acceptable, Table 2.

The work is limited as the data received was restricted and only CBCTs that were registered in the treatment position were suitable for review. Therefore, for some participants all CBCTs could be analysed and for others only a subset, as seen in Fig. 2, Fig. 3. For some participants weekly CBCTs were received and for some participants all CBCTs were received. Centres were provided with comprehensive support and guidance to facilitate the transmission of their data. Nevertheless, despite this assistance, it is recognized that transferring this specific radiotherapy data namely, registered CBCTs, remains resource-intensive and demands substantial support. This data is not routinely centrally collated in radiotherapy trials or routine practice. It was difficult to assess the trends over the course of the participant’s treatment due to data limitations. For example, adaption rates calculated only reflect the data that was suitable for review and not the true adaption rates if all the data was available. The second reviewer did not review all the plans selected; however, all selections between the first and second reviewer were concordant.

Adaptive radiotherapy is evolving with several options to re-plan daily. The PoD approach offers a cost-effective solution with established workflows that can be widely adopted, especially by centres lacking access to the latest online adaptive radiotherapy (oART) technology, broadening the benefit from improved treatment accuracy and consistency. The lessons learned from the during-accrual QA in the RAIDER trial, can inform the development and implementation of future QA methods for oART to reduce uncertainties in online plan selection.

In order to ensure consistency between different centres and different equipment RT QA will need to additionally consider the CBCT image quality, generated contours, the applied margins and the choice optimised plan. In radiotherapy trials it is generally the local principal investigator at a centre who undertakes or supervises the contouring and approves the plan. However, in real-time adaption these tasks may be designated to other staff groups, providing sufficient training has been undertaken [19, 20]. Centres typically implement their in-house training programs [19–21] to achieve the required quality standards in real-time adaption. In the context of a trial, RT QA can collaborate with these centres to ensure appropriate quality assurance. However, it is crucial to recognise that these efforts should not occur in isolation. The QA approaches outlined in this study, such as workshops, guidance, comprehensive reports and continuous feedback remain valuable tools to ensure a robust quality assurance process throughout the trial. By combining both in-house training and the recommended QA approaches, centres can achieve the highest levels of quality assurance for the successful implementation of the trial.

## Conclusion

During-accrual RT QA interventions had a positive impact on the plans selected and there was significant improvement in concordance between the online and offline plans selected from stage 1 to stage 2. This highlights the importance of during-accrual QA throughout a complex radiotherapy trial to maintain consistency and adherence to guidelines. By recognising the role of RTTs in adaptive radiotherapy trials, and providing them with the necessary training and support, they can successfully deliver adaptive treatment. Overall, this study advances the understanding of the importance of during-accrual IGRT QA in complex radiotherapy trials.

## Funding

The RAIDER trial (NCT02447549, CRUK/14/016) is funded by Cancer Research UK (C1198/A17533) and is supported by the Cancer Research UK-funded Clinical Trials and Statistics Unit at the Institute of Cancer Research (C1491/A15955, C1491/A25351, CTUQQR-Dec22/100004). Research at the Institute of Cancer Research is also supported by Cancer Research UK under programme C33589/A19727 and C33589/A28284. The UK National Radiotherapy Trials Quality Assurance (RTTQA) Group provided the radiotherapy quality assurance programme for the trial and is funded by the National Institute for Health and Care Research (NIHR).

## CRediT authorship contribution statement

**Amanda Webster:** Writing – original draft, Methodology, Investigation, Formal analysis, Data curation, Conceptualization. **Michael Francis:** Writing – review & editing, Conceptualization. **Hannah Gribble:** Writing – review & editing, Project administration, Data curation. **Clare Griffin:** Writing – review & editing, Methodology, Formal analysis. **Shaista Hafeez:** Writing – review & editing, Validation, Resources, Methodology, Conceptualization. **Vibeke N. Hansen:** Writing – review & editing, Resources, Methodology. **Rebecca Lewis:** Writing – review & editing, Supervision, Project administration. **Helen McNair:** Writing – review & editing, Resources, Methodology. **Elizabeth Miles:** Writing – review & editing, Supervision, Conceptualization. **Emma Hall:** Writing – review & editing, Supervision, Methodology, Funding acquisition, Conceptualization. **Robert Huddart:** Writing – review & editing, Resources, Methodology, Funding acquisition, Conceptualization.

## Declaration of competing interest

The authors declare the following financial interests/personal relationships which may be considered as potential competing interests: Robert Huddart reports grants received by their institution from Cancer Research UK, MSD and Roche, consultation fees from Janssen, Bristol Myers Squibb, Astellas, Roche and Nektar Pharmaceuticals, payment or honoraria from Roche, Bristol Myers Squibb and Merck, support for attending meetings and/or travel from MSD, Roche and Janssen, participation on a data safety monitoring board or advisory board for Biontech, Gilead and Merck and leadership or fiduciary role at Cancer Centre London, Parkside. Shaista Hafeez reports grants from NIHR Biomedical Research Centre at The Royal Marsden NHS Foundation Trust and The Institute of Cancer Research, non-financial support from Elekta (Stockholm, Sweden), personal fees and non-financial support from Roche, non-financial support from Merck Sharp & Dohme (MSD), outside the submitted work. EH reports non-financial support (study drug supplies) received by their institution from Astra-Zeneca and Bayer, outside the submitted work. EH also reports grants received by their institution from Cancer Research UK (within scope of submitted work and outside the submitted work) and Prostate Cancer UK (outside submitted work). Amanda Webster, Michael Francis, Hannah Gribble, Clare Griffin, Vibeke N Hansen, Rebecca Lewis, Helen McNair and Elizabeth Miles have no conflicts to declare
