# Supplementary material for: Impact of on-trial IGRT quality assurance in an international adaptive radiotherapy trial for participants with bladder cancer
Source: Radiother Oncol. 2024 Oct;199:110460. doi: 10.1016/j.radonc.2024.110460 (PMC11413485; doi:10.1016/j.radonc.2024.110460)
Supplement: Supplementary Data 1 [file mmc1.docx]

***Appendix A: RAIDER trial schema***

**
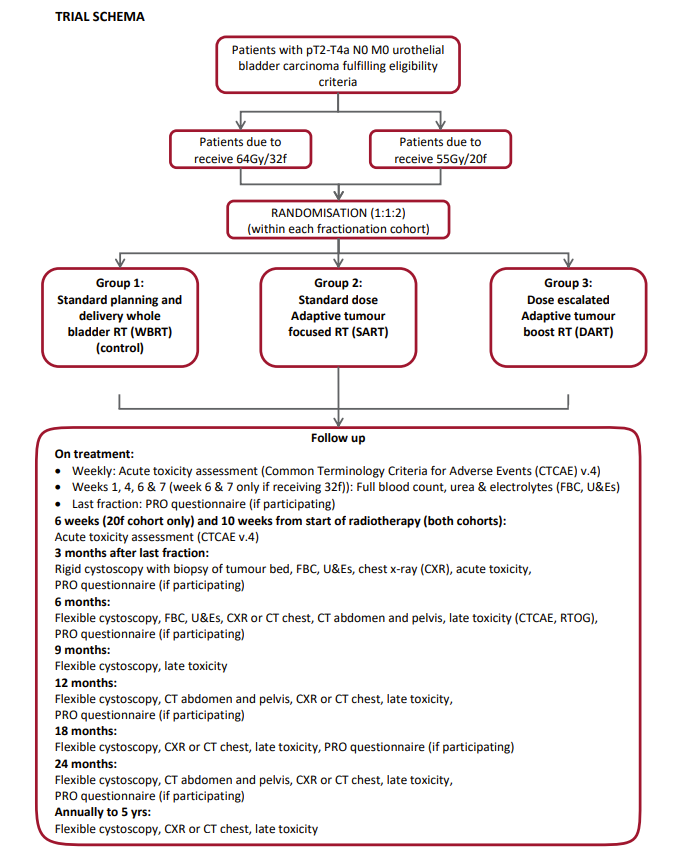
**

***Appendix B – Example of plan report***


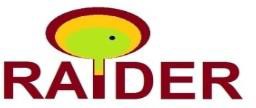

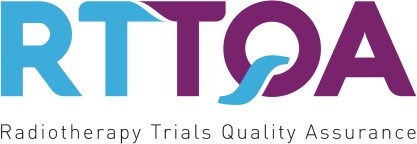


Retrospective Review of Treatment Data RXXXXX, Group X DART

| Centre: | XXXXX  XXXXX  XXXXX (RTTQA)  XX/XX/XXXX |
| --- | --- |
| Machines: |  |
| Reviewed by: |  |
| Date: |  |

# CBCT data submission

The following data was submitted for review:

- Fraction X,X,X,X,X (pre-CBCT)
- No post-treatment CBCTs were submitted

# CBCT data review

| **CBCT Date and description** | **Online**  **POD Decision** | **Comments** |
| --- | --- | --- |
| Fraction X CBCT  XX/XX/XXXX  Pre-treatment | Online plan selection detailed here | Assess:   1. Undertake match to bone first 2. Compare bladder volumes and check location of tumour. 3. Is the bladder volume on the CBCT comparable to the planning CT scan 4. If yes, immediately consider small plan. 5. If small not suitable consider medium. Is tumour coverage ok? 6. If the bladder volume is larger on the CBCT than the planning CT scan 7. Consider medium, if not suitable consider larger and assess OARs. Is tumour coverage ok? 8. Consider if too full and if the patient needs to be removed from the treatment couch 9. If the bladder volume is smaller on the CBCT than the planning CT scan 10. Consider small plan. Assess OARs. Is tumour coverage ok? 11. If too empty remove the patient from the treatment couch. |
| Fraction X  CBCT XX/XX/XX |  |  |

# Comments

Remark upon:

Concordance in matching between online and offline plan selections Determine if further reviews required

Highlight if no post-treatment CBCTs sent and the importance of this so that if can be determined if the patient's bladder fills or not while they are on the treatment couch

Highlight time taken to image match

Highlight if incorrect nomenclature used or not.

# Conclusion

Thank you for taking the time to submit this patient for review to RTTQA.

If you have any further questions please do not hesitate to contact: [XXXXX@XXXX.XXX](mailto:XXXXX@XXXX.XXX)
